# Cohort and Gender Differences in Stability Over Five Years Among Mexican-Origin Caregivers to Older Adults Living With Cognitive Impairment

**DOI:** 10.1177/01640275241310518

**Published:** 2025-01-08

**Authors:** Christian E. Vazquez, Sunshine Rote, Felipe Antequera, Alberto Cabrera, Phillip Cantu, Jacqueline L. Angel

**Affiliations:** 1 School of Social Work, 12329 The University of Texas at Arlington, Arlington, TX, USA; 2 Kent School of Social Work and Family Science, 5170 University of Louisville, Louisville, KY, USA; 3 LBJ School of Public Affairs, 12330 The University of Texas at Austin, Austin, TX, USA; 4 Graduate College of Social Work, 14743 University of Houston, Houston, TX, USA; 5 Department of Internal Medicine, Division of Geriatrics, 12338 University of Texas Medical Branch, Galveston, TX, USA

**Keywords:** cognitive impairment, caregiving, Mexican Americans, cohorts, gender

## Abstract

The current study examines cohort (i.e., Generation X, Baby Boomers, and Silent Generation) and gender differences in the stability of Mexican-origin family caregivers over time. The data comes from Mexican-origin caregivers to community-dwelling older adults living in the west and southwestern United States from the Caregiving Supplement to the Hispanic Established Populations for the Epidemiologic Study of the Elderly (2010/11–2016). Multinomial logistic regressions with interaction and predicted probabilities demonstrate that female caregivers are more consistent in their caregiving role over male caregivers in every cohort over the two time points (RRR = 0.67; 95% CI = [0.01–0.38]). Among men, predicted probabilities indicate more caregivers from Baby Boomer (.17) and Generation X (0.12) cohorts remain in the caregiver role compared to Silent Generation (.07). The findings suggest that gendered expectations may be changing as male caregivers increase in their provision of family care. Future research should consider generational diversity in Hispanic caregiving experience.

## Introduction

The Mexican-origin population is the largest racial and ethnic minority subgroup in the United States (U.S.) and this group is continuing to grow and age (U.S. Census Bureau, 2023; Funk & Lopez, 2022). Pre-COVID-19 pandemic, Hispanics had the highest life expectancy compared to other racial/ethnic groups in the U.S. (Arias & Xu, 2022). Despite having relatively long life expectancies, morbidity rates have not significantly decreased and rates of cognitive and physical impairment continue to grow for Mexican-origin Hispanics (Antequera et al., 2024). Combined with a decline in family size and longer lifespans with morbidity, Mexican-American families are left with an increasing old-age dependency ratio problem (Antequera et al., 2024). Traditionally, female spouses or daughters have taken on the role of family caregiver to older adults living with cognitive impairment – defined here by having dementia-related symptoms (Rodrigues et al., 2023; Rote et al., 2015). However, as demographic patterns shift, it is unclear what the stability of the caregiver role looks like when examined by gender and age cohort (i.e., Generation X, Baby Boomer, Silent Generation). The literature on family caregivers of those living with cognitive impairment has pointed to the negative effects of being a caregiver (Evans et al., 2017; Herrera et al., 2009) as well as the positive effects of continuity of care (Larsen et al., 2019), thus better understanding the ‘who’ that contributes to caregiver stability could provide information for intervening to support caregivers and care recipients who are likely to experience transition or help to delay transitions.

Older Hispanics living with cognitive impairment are estimated to rely on familial care for an average of 35.8 hours a week, care which would cost over $44,000 a year if provided by professional sources (Cantu et al., 2024). Thus, the role of caregivers to older adults living with cognitive impairment within Mexican-origin families will continue to be a topic not only impacting a large group but also U.S. social service and health resources. Aging in place is considered the best option for those living with cognitive impairment (Samus et al., 2018, 2020), though the success of aging at home can be complicated by caregiver instability (Dukhovnov & Zagheni, 2015; Uccheddu et al., 2019; Wawrziczny et al., 2021). A better understanding of caregiver stability in the caregiver role can help provide valuable insights for healthcare providers, insurers, and policymakers to offer services to better support caregivers. There is a lack of studies examining caregiver stability within care for those with cognitive impairment, even more so with U.S.-based Mexican-origin families. Using two time points over a 5-year period, the current study focuses on gender and cohort effects in caregiver stability: Generation X (ages 31 to 45 at baseline), Baby Boomers (ages 46 to 64 at baseline), and the Silent Generation (ages 65 to 82 at baseline).

The literature suggests that caregiving transitions are related to a number of reasons, such as progression of cognitive impairment that leads to more needs than a family caregiver can provide, caregivers’ economic and stress-related issues and health challenges, strain of caregiver-care recipient relationship, or a lack of social support (Angel et al., 2016; Charenkova, 2023; Dombestein et al., 2020; Dukhovnov & Zagheni, 2015; Falcão & Paulson, 2022; Roberto & Savla, 2022; Uccheddu et al., 2019; Venters & Jones, 2021; Wawrziczny et al., 2021). For Mexican-origin family caregivers, stressors such as financial strain, emotional distress, lack of respite care options, and limited access to support services have been reported to impact overload and burnout though do not necessarily lead to transition out of the role as other factors may moderate their impact (Evans et al., 2017; Herrera et al., 2009). For example, Gelman (2014) found some protective factors of staying in the caregiver role such as familism (the strong identification and attachment of individuals to their families) and filial piety (attributes of respecting, caring for, and loving the parents) for Hispanics. It was suggested that these factors strengthened the caregiver-care recipient relationship. Though the literature from cross-sectional studies on factors related to transition out of the care role is helpful, there is still little evidence on how stability is impacted by individual caregiver factors such as gender and age group over time, which can be paired with the literature above to inform appropriate intervention for Mexican-origin caregivers.

### Gender-Specific Care Expectations

Gendered expectations of care continue to exist across the globe with most studies indicating women are more often the primary caregivers for older family members compared to men (Chappell et al., 2015; Day et al., 2014; Dukhovnov & Zagheni, 2015; Rodrigues et al., 2023; Rote et al., 2015). The literature suggests that men who become caregivers are more likely to be paid, suggesting they feel they have a choice where women may transition into this role unpaid due to gendered expectations, and this is true for Hispanic populations as well (Baik et al., 2024; Rote et al., 2019). The burden of gendered expectations may be more pronounced for racial and ethnic minority women (Cohen et al., 2019). For Hispanics specifically, these expectations appear to be influenced by structural as well as cultural factors such as marianismo—expectations of feminine sacrifice. However, a recent systematic review suggests that differences among caregivers are complex and that there is no clear gendered finding relating to burden or stability (Xiong et al., 2020). The current study builds on these works to further quantitatively examine the effect gender has on the probability of staying in the caregiver role longitudinally.

### Generational Differences

The studies on gender also intersect with the studies on generational caregiving. Not only are women more often providing care, but that effect is not altered by generational status (Dukhovnov & Zagheni, 2015; Rigby et al., 2019; Rodrigues et al., 2023; Venters & Jones, 2021; Wawrziczny et al., 2021). These findings may reflect the “sandwich” generation phenomenon in which adult children are caring for both their parents and their own children (Rapp et al., 2023). Changing of values from generation to generation may play a significant role in shaping caregiving experiences and practices (Angel & Angel, 1997; Becerra & Shaw, 1984; Clark & Huttlinger, 1998; Day et al., 2014). For example, older generations may hold traditional beliefs regarding filial piety and view caregiving as a duty that should be fulfilled within the family and by women (Becerra & Shaw, 1984). Generational differences may apply particularly to Mexican-origin families partly because of a combined sense of filial and moral obligation (Aranda & Knight, 1997; Mendez-Luck & Anthony, 2016). The current study builds on literature related to the unique life course trajectories of each cohort to apply an age cohort lens to better understand the ‘who’ in caregiver stability.

The unique life course trajectories of Mexican Americans, their cultural identities, and the interplay of gender and generation shape the expectation of care and the care for older adults (Torres-Gil, 2022). The heterogeneity in life course experiences by different generations motivate an analysis of care giving relationships in late life. The oldest old of today, people born in the 1930’s are cared for by adults who were part of the Silent Generation, Baby Boomer, and Generation X, and these adults face different gender, cultural, and work expectations concurrent with the values of their cohort. For example, caregivers who were born during the Silent Generation (prior to 1945) may have experienced their migration to the U.S. during the Bracero Period (1942–1964), a period of immigration marked by bilateral agreement between the U.S. and Mexico to import cheap labor to the U.S. (Bean, 1997). Conversely, Generation X (born between 1965 and 1980) caregivers may be more likely to be U.S.-born children of the adults they care for. Caregivers from the Baby Boomer Generation (1946–1964), straddle the lines of the immigrant trajectories, being drawn from both U.S. and Mexican-born experiences. The immigration contexts of the different generations are exemplary of the varied lives of these generations. Mexican Americans’ place in American public life has shifted over generations, with successive cohorts having greater access to education, especially among women, which has increased the workforce opportunities (Blancero et al., 2018). Differences in immigration policy, educational attainment, marriage, living arrangement, and fertility across generations have resulted in vastly different life course contexts for caregivers from each generation. Adults from Generation X would have had more opportunities to work outside the home, delay marriage, and complete more years of education compared to caregivers from the Silent Generation. However, these new opportunities present challenges for Mexican Americans as the calculus of care has become more complicated, weighing cultural expectations and gender roles against competing obligations outside the home and family (McDermott & Mendez-Luck, 2018).

Studies using longitudinal data have only recently emerged to be able to answer questions about patterns in caregiver stability in the U.S. There are three notable studies in this area. Spillman et al.’s (2020) study, which uses two time points over four years, suggests that change over time in the person identified as the primary caregiver is common. Additionally, they suggest that more caregiver transitions lead to poor outcomes for the care recipient and a poorer experience for the caregiver, compared to a stable caregiver-care recipient relationship. Allen et al.’s (2012) study, which uses three time points over 6 years, found that more than half of primary family caregivers transitioned over 2 years to a different primary caregiver. Further, Szinovacz and Davey (2013) use multiple time points over 8 years and indicate that among adult child caregivers providing help to their parents, more than one quarter of adults identified as the primary caregiver were replaced by a sibling, and two thirds of networks added or dropped sibling caregivers over a 2-year period. Though nuclear family size is declining among Mexican Americans, this group may still have more collective caregiving and more frequent transitions, yet further research is needed to understand stability in this group as demographic patterns shift (Evans et al., 2017).

This exploration of transitions out of caregiving uses the lens of cohort and gender differences to explore the stability of Mexican-origin family caregivers. In the current study, the terms cohort and generation are used interchangeably when discussing the three groups mentioned above. The small amount of literature on stability and the intersection with cohort and gender findings warrants further examination of this topic with an understudied group. While more is known about factors precipitating caregiver transition out of their role (Angel et al., 2016; Charenkova, 2023; Dombestein et al., 2020; Falcão & Paulson, 2022; Roberto & Savla, 2022; Uccheddu et al., 2019; Venters & Jones, 2021; Wawrziczny et al., 2021), little is known about the ’who’ in the stability of caregiver-care recipient relationship over time. This is an important aspect to understand because it could provide information for intervening to support caregivers in their transition or help delay transitions if that is what might be best for the family.

*The overarching research question is*: Are caregiver gender and cohort associated with caregiver stability using two time points among U.S.-based Mexican-origin caregivers to older adults living with cognitive impairment?

## Data

The study employs two time points of data, over a 5-year period, on Mexican-origin caregivers to community-dwelling older adults in the U.S. from the Caregiver Supplement of the Hispanic Established Populations for the Epidemiologic Study of the Elderly (HEPESE CG, 2010/11–2016). The HEPESE is representative of Mexican Americans 65 years and older living in five southwestern states: Arizona, California, Colorado, New Mexico, and Texas (Black et al., 1998). Data collection began in 1992/1993 with 3050 older adults and continued every 2-3 years with a replenishment sample added in 2005. In 2010 and 2011, older adults participating in the HEPESE (*N* = 1078) were asked for contact information for the person they rely on most for help. This led to interviews with 925 primary caregivers, of which 817 were family caregivers of the older adult. In 2016, older adults participating in the HEPESE were asked again for contact information for the person they rely on most for help. Out of the original 817 care recipients 87 were lost to follow-up or refused to participate, which led to data on 730 care recipients. Between baseline and follow-up, 415 care recipients died and at follow-up 315 caregivers were interviewed. The baseline data for the 415 cases lost to mortality were still eligible for inclusion in the analysis. As the current analysis is focused on caregiver cohort and stability, we drop caregivers from the Greatest Generation (*n* = 14) and Millennial cohorts (*n* = 22) due to their limited sample size and include 667 family caregivers to older adults with full information on all key variables at baseline (*n* = 27 missing on key variables). At baseline, most of the caregivers were adult children (about 80%) compared with other relatives (spouses (6%), grandchildren (4%), and other relatives (10%)).

In auxiliary analyses, there were significant differences in the study variables for caregivers with complete information on baseline characteristics and missing between time points (*n* = 79) and the final analytic sample (*n* = 667). Caregivers who were lost to follow-up were more likely to complete the baseline interview in English (vs. Spanish) and provide care for a care recipient with lower IADL and ADL disability relative to caregivers who remained in the study. An important note is that while the study period covers two time points over 5 years, the HEPESE did not specifically ask about transitions within the five years between baseline to follow-up and we cannot determine whether there were changes over 5 years and back to the same caregiver. This is noted as a limitation further below. Institutional Review Board approval was waived for this study as it is an analysis of secondary data.

## Measures

### Dependent Variable

*Caregiver Stability* was assessed by measuring the change in the caregiver between baseline and follow-up (coded 0 = same caregiver at both time points, 1 = new caregiver at follow-up, 2 = care recipient died between time points).

*Caregiver cohort/generation* is measured by assessing the caregiver’s year of birth and is divided into the following categories: Silent Generation (*n* = 129; 1928–1945; ages 65 to 82 at baseline), Baby Boomer (*n* = 435; 1946–1964; ages 46 to 64 at baseline), and Generation X (*n* = 103; 1965–1980; ages 31 to 45 at baseline). If the caregiver did not report their year of birth but reported their age at baseline (*n* = 8), age was used to determine caregiver cohort/generation.

Caregiver background factors included caregiver *gender* (male vs. female), *Spanish* language interview (Spanish vs. English), whether the caregiver is an *adult child* of the care recipient (adult child vs. other relative), and self-reported *household income* which was coded to distinguish among caregivers in the top half of the distribution (e.g., $20,000 to $50,000 and over), the bottom half of the distribution ($0 to $19,999), and those missing on income. We also control for *caregiver self-rated health* (excellent to good vs. fair to poor). Care recipient background factors include *age* (range: 79–102) as a continuous variable, *gender* (male vs. female), *nativity* (U.S.-born vs. born in Mexico), and *household size* (lives alone, lives with 1-2 people, and 3 or more). *In terms of care recipient health and need for assistance,* we include high level of care recipient need for help with activities of daily living (ADLs) (3 or more ADLs) and instrumental activities of daily living (8 or more IADLs). ADLs include: bathing, dressing, toileting, transferring bed/chair, walking, and eating (mean (M) = 2; standard deviation (SD) = 2 before recoding). IADLs include: managing budget, shopping, cooking, managing medications, using a telephone and looking up numbers, light housework, and driving or using public transportation (M = 6; SD = 3 before recoding).

*Neuropsychiatric symptoms related to dementia* (Neuropsychiatric Inventory, NPI; Cummings, 1997; 2020) are caregiver-reported and includes the frequency of 12 symptoms expressed by the care recipient in the past month such as wandering, agitation, and aggression. The NPI is calculated by multiplying the frequency and severity of each of these symptoms (none = 0, mild = 1, moderate = 2, severe = 3) by the distress resulting from these symptoms (not at all = 0, minimal = 1, mild = 2, moderate = 3, and severe = 4) (ranges 0 to 55; M = 7 SD = 9). The recoding of these variables was data-driven comparing the top quarter of the distribution (9 or more = 1) to the bottom three-quarters of the distribution (8 or less = 0) to identify high level of need for dementia-related support and care.

## Analytic Strategy

**Figure 1.**
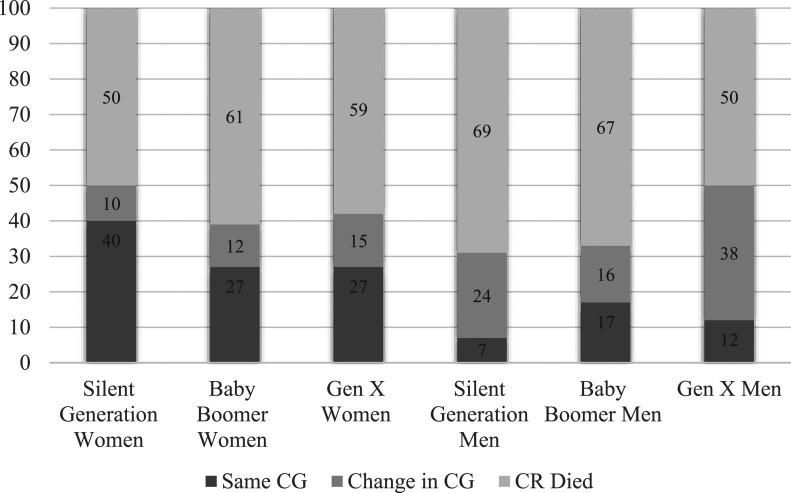
Predicted probabilities of caregiver transitions over five years from interaction effects of caregiver gender by caregiver cohort (*N* = 667).

Complete case analysis was used for all analyses. In the first step of the analysis, we present descriptive statistics of the study variables by gender (Table 1). In the second step, we present relative risk ratios with 95% confidence intervals (CIs) from multinomial logistic regression analyses focusing on gender and cohort, controlling for interview language, caregiver relationship to care recipient (adult child vs. other relative), household income, caregiver self-rated health, care recipient gender, care recipient born in the U.S., care recipient age, household size, instrumental activities of daily living hours, activities of daily living hours, care recipient neuropsychiatric symptoms related to dementia, and the interactive effects of gender*cohort (Table 2 and Table 3). In step 3, we produce predicted probabilities for caregiver stability based on the analysis in step 2 (Figure 1). All analyses were performed using STATA18.

**Table 1.** Sample Description by Gender, Caregiver Supplement to the Hispanic Established Populations for the Epidemiologic Study of the Elderly (HEPESE CG, 2010/11–2016), *N* = 667.

	Female caregivers (*n* = 484)	Male caregivers (*n* = 183)	χ^2^ *p* value
Caregiver transitions			<.001
Same caregiver	31%	18%	
New caregiver	13%	22%	
Care recipient mortality	56%	60%	
Caregiver cohort			n/s
Silent generation (ages 65 to 82 at baseline)	20%	16%	
Baby boomer (ages 46 to 64 at baseline)	65%	66%	
Generation X (ages 31 to 45 at baseline)	14%	18%	
Caregiver background and health			
Spanish interview	64%	64%	n/s
Adult child caregiver	88%	76%	<.01
Household income			n/s
≤$19,999	45%	40%	
≥$20,000	46%	49%	
Missing	9%	11%	
Good to excellent self-rated health	57%	58%	n/s
Care recipient background and health			
Female	64%	66%	n/s
U.S.-born	56%	55%	n/s
Care recipient age (M(SD), range 79–102)	87 (4)	87 (4)	n/s
Care recipient household size			<.01
Lives alone	25%	23%	
Lives with others (1-2 people)	49%	61%	
Lives with others (3 or more)	27%	16%	
Care recipient IADLs (8+)	43%	38%	n/s
Care recipient ADLs (3+)	34%	31%	n/s
Care recipient NPI (9+)	29%	27%	n/s

*Note.* Note. IADLs = instrumental activities of daily living. ADLs = activities of daily living NPI = neuropsychiatric symptoms related to dementia. For caregiver relationship to care recipient, the categories are adult children versus spouses, grandchildren, and other relatives. χ^2^ = chi-squared statistic. n/s = not significant.

**Table 2.** Multinomial Logistic Regression Results of Caregiver Transitions Over Five Years in the HEPESE, *N* = 667.

Base category (same caregiver)	New caregiver	Care recipient mortality
Female caregiver	0.29 [0.16–0.52]	0.45 [0.28–0.73]
Caregiver cohort		
Baby boomer	1.24 [0.59–2.63]	1.48 [0.84–2.61]
Generation X	2.21 [0.93–5.22]	1.43 [0.70–2.91]
Spanish interview	0.85 [0.49–1.49]	0.69 [0.45–1.06]
Adult child caregiver	0.58 [0.29–1.14]	0.81 [0.47–1.41]
Household income		
<$20,000	0.94 [0.54–1.63]	0.96 [0.63–1.46]
Missing	2.99 [1.16–7.67]	2.23 [0.99–5.02]
Good to excellent self-rated health	0.55 [0.32–0.94]	0.80 [0.53–1.22]
Female care recipient	0.82 [0.48–1.41]	0.61 [0.41–0.94]
U.S.-born care recipient	1.41 [0.83–2.39]	1.11 [0.75–1.66]
Care recipient age	1.05 [0.97–1.13]	1.12 [1.06–1.19]
Care recipient household size		
1 to 2	0.59 [0.30–1.14]	0.47 [0.28–0.78]
3 or more	0.66 [0.30–1.46]	0.57 [0.31–1.05]
Care recipient IADLs 8+ hours	1.54 [0.77–3.10]	2.12 [1.27–3.55]
Care recipient ADLs 3+ hours	0.96 [0.46–2.03]	1.97 [1.15–3.37]
Care recipient NPI 9+ symptoms	0.81 [0.41–1.62]	2.26 [1.41–3.61]
Intercept	0.05 [0.00006–33.96]	0.0002 [0.00001–0.04]
χ^2^	158.90	

*Note.* Note. Relative risk ratios with 95% confidence intervals. Reference categories: male caregiver, Silent generation, English interview, not adult child caregiver, ≥$19,999, poor to fair self-rated health, male care recipient, non-U.S.-born care recipient, living alone, low IADL care, low ADL care, low NPI. IADLs = instrumental activities of daily living. NPI = neuropsychiatric symptoms related to dementia. χ^2^ = chi-squared test of significance.

**Table 3.** Multinomial Logistic Regression Results of Caregiver Transitions Over Five Years in the HEPESE by Gender and Cohort Interactions, *N* = 667.

Base category (same caregiver)	New caregiver	Care recipient mortality
Female caregiver	0.67 [0.01–0.38]	0.12 [0.02–0.59]
Caregiver cohort		
Baby boomer	0.27 [0.48–1.52]	0.39 [0.08–1.96]
Generation X	0.88 [0.13–6.12]	0.40 [0.06–2.56]
Female*Baby boomer	7.08 [1.08–46.32]	4.62 [0.86–24.94]
Female*Generation X	2.64 [0.30–23.17]	4.42 [0.60–32.32]
Intercept	0.10 [0.0001–90.10]	0.0007 [0.00001–0.14]
χ^2^	166.14	

*Note.* Note. Relative risk ratios with 95% confidence intervals. Reference categories: male caregiver, Silent generation. Controlling for all other variables in Table 2: interview language, caregiver relationship to care recipient (adult child vs. not), household income, caregiver self-rated health, care recipient gender, care recipient born in the U.S., care recipient age, household size, instrumental activities of daily living hours, activities of daily living hours, and care recipient neuropsychiatric symptoms related to dementia. χ^2^ = chi-squared test of significance.

## Results

Table 1 describes the sample by caregiver gender. There were clear gender differences in caregiver stability, with more female (31%) than male (18%) caregivers remaining in the role at both time points (χ^2^(2) = 16.47; *p* < .001). Most caregivers were part of the Baby Boomer Generation, followed by the Silent Generation, then Generation X, and there were no statistically significant gender differences in the caregiver cohort. Most caregivers completed the interview in Spanish, and were adult children of the care recipient, though more men (88%) than women (76%) were adult children (χ^2^(2) = 11.11; *p* < .01). Less than half of the caregivers had low household incomes and just over half of caregivers reported their health as good to excellent. For the care recipients, most were US-born older adults, female (65%), and over 80 years old. Most caregivers cared for older adults living in households of 1-2 people; however, over a quarter were providing care for older adults who were living alone. Caregivers were providing care for older adults with high levels of IADL disability and ADL disability and 28% of caregivers reported a 9 or more on the NPI scale, which reflects care recipient’s level of need for dementia-related symptoms.

Table 2 presents results from multinomial logistic regression analyses with caregiver gender and caregiver cohort predicting caregiver stability. Similar to the bivariate results, the regressions show women were more likely than men to be in their caregiving role at the second time point. For ease of interpretation, we report the probability of change calculated from the relative risk ratios. For example, women caregivers had a 71% (95% CI = [0.16–0.52]) lower relative risk of having a new primary caregiver take over and a 55% (95% CI = [0.28–0.73]) lower relative risk of care recipient death across the two time points relative to men. When caregivers reported good to excellent self-rated health, compared to those reporting poor to fair, there was a 45% lower relative risk of transition to a new caregiver (RRR = 0.55, 95% CI = [0.32–0.94]). Factors related to caregiver transition due to care recipient mortality include the care recipient being female (RRR = 0.61, 95% CI = [0.41–0.94]), older care recipient age (RRR = 1.12, 95% CI = [1.06–1.19]), care recipient household size of 1–2 people compared to none (RRR = 0.47, 95% CI = [0.28–0.78]), providing help for 8 or more IADLs care compared to less (RRR = 2.12, 95% CI = [1.27–3.55]), providing help for 3 or more ADLs care compared to less (RRR = 1.97, 95% CI = [1.15–3.37]), and a NPI score of 9 or more compared to less (RRR = 2.26, 95% CI = [1.41–3.61]).

In the next step, we assessed whether the association between gender and caregiver stability was conditional on caregiver cohort. In this model, we added an interaction term of gender*cohort and controlled for the background factors in the previous model. The only statistically significant interaction result in this model was the interaction between female and Baby Boomer Generation (RRR = 7.08, 95% CI = [1.08–46.23]), which indicated female Baby Boomers had a higher relative risk ratio of being in caregiver role at both time points, compared to males from the Silent Generation (see Table 3). Follow up tests were examined in step 3.

The predicted probabilities from the multinomial logistic regression model are presented in Figure 1. Figure 1 demonstrates that there was more consistency in who the caregiver is at time point two for women caregivers relative to men. For example, the predicted probability of remaining in the caregiver role for women in the Silent Generation cohort was 0.40, for women in the Baby Boomer cohort was 0.27, and for women in the Generation X cohort was 0.27. For men, the predicted probability of being in the caregiver role after time point two was .07 for caregivers in the Silent Generation cohort, 0.17 for caregivers in the Baby Boomer cohort, and 0.12 for caregivers in the Generation X cohort.

## Discussion

The current study examines cohort (i.e., Generation X, Baby Boomers, Silent Generation) and gender (female vs. male) differences in the stability of Mexican-origin primary caregivers of those living with cognitive impairment. Better understanding the ‘who’ that contributes to caregiver stability could provide information for intervening to support caregivers or help delaying transitions. This study suggests that women caregivers are more likely than men to remain in the caregiver role over time, but our findings among generations are more complex.

Each set of analyses and the predicted probabilities across all cohorts showed higher probabilities of continuity in the primary caregiver role for women than for men. For women, stability was most associated with the oldest generation, Silent, followed by Baby Boomers, and Generation X being associated with the most instability. This relationship for Generation X women may be explained by several factors. For example, Generation X may be experiencing the effects of being a “sandwich” generation where they are being pulled in many directions providing care for parents and children of their own (Rapp et al., 2023). Additionally, Generation X, at the time of data collection, would be at the height of professional careers that contribute to time constraints and decision-making about when and who to care for (Blancero et al., 2018). In the current study findings, Generation X and Baby Boomer men were more likely to remain in a caregiver role than Silent Generation men and Generation X women were less likely than Silent Generation women, though women were still more stable caregivers than men in every generation. Thus, Generation X may represent a point in time where a generational shift begins related to women’s stability in providing care which was much stronger in older cohorts. Generation X men’s lower stability compared to Generation X women may reflect continued gendered societal expectations with respect to career.

For men, the patterns were different, compared to women by generation, with Baby Boomer men the most consistent in their role. Generation X also had higher stability than the Silent Generation. Silent Generation men may also be dealing with their own health concerns as they are older and this may explain the transition to someone else, though it is not clear if this responsibility transitions to other men or to women (Rote et al., 2019). However, we are not able to control for caregiver self-rated health in the current study, and examining this in future work is part of the next step. Generation X men may be facing similar challenges to Generation X women related to “sandwich” caregiving or competing work demands. Similar to health status, employment was not available for all caregivers, examining employment status or transitions along with caregiving over time is another important next step.

There may be an opportunity for intervention with Generation X. The findings show there is twice as much instability for men compared with women. Though men and women from Generation X may be facing similar challenges, the findings highlight the continuation of a relationship that situates the caregiver role onto women. The norm that men’s work obligations come before women’s obligations may be persisting. Qualitative work also suggests that male caregivers struggle in the caregiver role due to unexpected expectations (Rote et al., 2019). Unexpected expectations can take the form of expectations related to the type of caregiving (e.g., bathing) that men may not be willing to take on, leading them to transition out of the role. It may be possible that men expect to provide spousal care but not parental or grandparent care, which would affect the higher odds of instability in the current study. The relationships found here point to gender diversity in care possibly remaining consistent or increasing with time. Research and interventions may want to follow Generation X to understand how to provide supports around a group that may bring changes in consistency to the familial caregiver role. Additional evidence for focusing on Generation X specifically among Hispanics is that studies have started to show more gender parity and a general increase in Millennial caregivers (Flinn, 2018). Thus, Generation X could be a potentially important group to target with more inconsistency currently displayed amongst Hispanics.

It is also important to note that historical movements, impacting Generation X, have contributed directly to changes in gender norms related to labor force participation, education, marital disruption, and potentially indirectly to changes in the amount of time family members devote to caregiving. Increases in education for women over subsequent cohorts may change the gender-based norms of caregiving over time (McDermott & Mendez-Luck, 2018). For example, increases in education have been linked to reductions in familial care and it is possible that expansion of education for Generation X women compared to the Silent Generation may provide outside incentives for women to transition out of caregiving roles. While other studies have found that across generations, Hispanics continue to provide more familial care than non-Hispanics (Miyawaki, 2016), the current findings suggest there could be potential changes in expectations for female caregivers of more recent birth cohorts based on there being lower odds of remaining in the caregiver role for this group.

Additionally, while we posit that changes in the role of Mexican Americans in American public life has changed dramatically over the last century, we are unable to speak to the role of important Mexican-American political movements in this study (Torres-Gil, 2022). Especially among those providing care in California, the Chicano Movement of the last half of the 20th century may have important implications for how women situate themselves within the family and provide care. Our findings indicate that the gender*cohort relationship warrants continued attention, particularly as the Silent Generation ages out and Millennials become caregivers, especially in dynamic longitudinal models considering age, period, and cohort effects.

## Implications

Caregiving for a family member living with cognitive impairment has many implications for the caregiver’s life, creating challenges in areas such as mental health, physical health, and workforce participation. These factors may be more pronounced during different phases of life and based on gender. Yet even as womens’ role in the workplace impacts their availability for long-term care, they show greater stability in the caregiving role than men. At the same time, more men are becoming caregivers and staying in this role longer. Thus, perhaps a lessening of this burden of choice for women is on the distant horizon. This could lead to the need to support women who may have conflicted feelings about choosing their own needs over caregiving, as well as supporting men who have not historically been in this role, especially for long periods of time. Even though men may play an increasingly active role in caregiving, like women they experience the same strains which may take a toll on physical, psychological, and financial wellbeing.

Stability can be both positive and negative, depending on the situation, though continuity of quality care is ideal. The current study findings indicate that, particularly when men are caregivers, there may be continuity challenges. Given the challenges of balancing the caregiver’s needs with caregiving, efforts can be made to create more seamless caregiving experiences that might sustain longevity in this role if that is what is best. Creating improved experiences may need to be tailored to the caregiver’s life phase. Older caregivers such as spouses who are out of the workforce may have health needs of their own that require attention (i.e., they may be neglecting their own health). Where younger caregivers may need support to balance their competing responsibilities in the workforce, with their own families, and mental health (i.e., there me be practical time constraints leading to significant stress).

## Limitations

Although the study has many strengths, including a wealth of data on an understudied prioritized caregiver population, and longitudinal data, a small sample size limits detailed analyses. The sample was relatively small due to attrition over time, though the estimates were robust. The care recipients in the current study were of advanced age (mean age 87), and there may be clearer gender and cohort differences in caregiver stability earlier in the care trajectory when care recipients are younger and starting to need care. Another limitation is that while we focused on the change in primary caregiver, the dataset does not provide much information on the care network available and possible transitions from one caregiver back to the primary caregiver within the 5 years from baseline to follow-up. For example, we do not know whether secondary caregivers are supporting the older adult to a similar but lesser extent or whether the caregivers in this sample are the only ones providing care. Additionally, the data did not provide much information on labor market participation, which is an important factor here. Further exploration on this topic is warranted, with perhaps a focus on labor participation’s effect on caregiving stability across ages. Lastly, we are unable to control for caregiver age given that the cohort/generation variable is based on caregiver age, and this would lead to multicollinearity in the models. However, we acknowledge that further longitudinal models using a dynamic cohort analysis would allow for a better exploration in age and cohort effects and untangling the two. Documenting these differences across cohorts is the first step before more dynamic models are specified.

## Conclusion

The current study puts forth evidence of stability of familial caregiver to older adults living with cognitive impairment and can serve as a reference for future studies that include Millennials and Generation Z. There appears to be variation in the patterns of caregiver stability across gender and cohort. The reasons for this may be different for each life phase. For example, Generation X males and Silent Generation males have different needs and responsibilities at their respective ages. It will be important to observe how patterns continue to vary to understand the ways in which to support caregivers and care recipients. Better understanding the ‘who’ in caregiver stability can help inform interventions as our population continues to age and the need for care of those with cognitive impairment becomes higher.
